# Determinants of Motion Sickness in Tilting Trains: Coriolis/Cross-Coupling Stimuli and Tilt Delay

**DOI:** 10.3389/fneur.2017.00195

**Published:** 2017-05-15

**Authors:** Giovanni Bertolini, Meek Angela Durmaz, Kim Ferrari, Alexander Küffer, Charlotte Lambert, Dominik Straumann

**Affiliations:** ^1^Department of Neurology, Zurich University Hospital, Zurich, Switzerland; ^2^Institute of Pharmacology and Toxicology, University of Zurich, Zurich, Switzerland; ^3^Neuroscience Center, University and ETH Zurich, Zurich, Switzerland; ^4^Department of Neurosurgery, Zurich University Hospital, Zurich, Switzerland

**Keywords:** motion sickness, tilting trains, cross-coupling, otolith, semicircular canal, self-motion perception

## Abstract

Faster trains require tilting of the cars to counterbalance the centrifugal forces during curves. Motion sensitive passengers, however, complain of discomfort and overt motion sickness. A recent study comparing different control systems in a tilting train, suggested that the delay of car tilts relative to the curve of the track contributes to motion sickness. Other aspects of the motion stimuli, like the lateral accelerations and the car jitters, differed between the tested conditions and prevented a final conclusion on the role of tilt delay. Nineteen subjects were tested on a motorized 3D turntable that simulated the roll tilts during yaw rotations experienced on a tilting train, isolating them from other motion components. Each session was composed of two consecutive series of 12 ideal curves that were defined on the bases of recordings during an actual train ride. The simulated car tilts started either at the beginning of the curve acceleration phase (no-delay condition) or with 3 s of delay (delay condition). Motion sickness was self-assessed by each subject at the end of each series using an analog motion sickness scale. All subjects were tested in both conditions. Significant increases of motion sickness occurred after the first sequence of 12 curves in the delay condition, but not in the no-delay condition. This increase correlated with the sensitivity of motion sickness, which was self-assessed by each subject before the experiment. The second sequence of curve did not lead to a significant further increase of motion sickness in any condition. Our results demonstrate that, even if the speed and amplitude are as low as those experienced on tilting trains, a series of roll tilts with a delay relative to the horizontal rotations, isolated from other motion stimuli occurring during a travel, generate Coriolis/cross-coupling stimulations sufficient to rapidly induce motion sickness in sensitive individuals. The strength and the rapid onset of the motion sickness reported confirm that, even if the angular velocity involved are low, the Coriolis/cross-coupling resulting from the delay is a major factor in causing sickness that can be resolved by improving the tilt timing relative to the horizontal rotation originating from the curve.

## Introduction

The growing demand of high-speed land transport of goods and passengers in the last 30 years led to reinforcing the offer of fast trains, capable of maintaining high speed in curves by tilting the car bodies. The tilting mechanism compensates for the centripetal acceleration during turns by bringing the vertical axes of the cars closer to the gravito-inertial force vector. As a result, the trains can run faster, and the lateral thrusts of centrifugal force on the passengers during turns are decreased. Although, at first glance, this can be expected to increase comfort, many passengers in tilting trains develop symptoms of motion sickness (1, 2). Motion sickness (3–7) is a syndrome elicited in otherwise healthy subjects, whenever they are exposed to combination of motion stimuli at variance with those expected by the brain during our natural self-propelled motion. Its symptoms vary from simple stomach awareness to severe nausea and vomiting, but include also drowsiness, apathy, and irritability (3, 4, 8). These symptoms, besides decreasing the comfort, can also have consequences on the performance of workers exposed to passive motion (e.g., co-drivers, navigators, service personnel, and others), possibly affecting them without their awareness (sopite syndrome) (9, 10). Several years of research highlighted many combinations of motion stimuli that can induce motion sickness (3–5). In everyday life, these motion stimuli occur mainly, but not exclusively, when an individual is subject to a motion that he or she does not actively control (passive motion). For this reason, motion sickness is often associated with transport systems (car, boat, planes) as any of them induces unnatural motion stimuli (5, 11, 12).

Traveling on a tilting train provides a complex combination of passive motion stimulation. A detailed understanding of the mechanism underlying motion sickness in tilting trains is essential because only such knowledge allows developing technical solutions for the problem (13). Previous research, following accurate recording of the linear and angular motion of the cars, suggested that three components of train motion can possibly cause motion sickness: the jitters of the car, the centrifugal acceleration during the curves, and the tilt of train itself. The jitters show peaks at 0.5 and 1.7 Hz both in roll rotation and lateral linear accelerations (14). The latest are often pointed out as the main cause of motion sickness evoked by road transport, but the critical frequencies for these stimuli to evoke motion sickness was found to be around 0.16–0.2 Hz (15–18), suggesting a relative minor role of these stimuli in the train. The centrifugal forces also generate lateral accelerations. These accelerations are present in any train entering a curve. In the tilting trains, the magnitude of centrifugal acceleration is larger due to the higher velocity allowed by the tilt of the cars, but the actual amount of lateral acceleration on the passenger is also decreased on the tilts as they align the body vertical axis to the gravito-inertial vector. Various studies showed the nauseogenic role of the lateral linear acceleration in actual and simulated conditions, in particular if combined with roll tilts (1, 13, 19–22). Accordingly, motion sickness of passengers in tilting trains can be reduced by decreasing the angle of the compensatory roll tilt (2, 14, 23). The usefulness of this approach is limited because the velocity of trains on curves has to decrease, which negates the purpose of the tilt. The tilt of the train while in curves, finally, provides a typical nauseogenic stimulus, known as Coriolis/cross-coupling. Such stimulus occurs when, during an ongoing rotation around a space fixed Earth-vertical axis, a person sudden tilts the head around an Earth-horizontal axis (24). In the head reference frame the change direction of the gravity vector describing the re-orientation with respect to gravity from the subject point of view is not accompanied by the correct re-orientation of the sensed rotation axis that will keep it aligned to gravity as it actually is (remember that the ongoing rotation is around a space fixed Earth-vertical axis). This occurs because the semicircular canals behaves like a high-pass filter of the input velocity and the sensed velocity of the ongoing rotation decay according to an approximately exponential function determined by the property of the semicircular canals, the neural transduction, and the different step of central processing (25–27). If there is a delay between the onset of the first rotation and the tilt, the velocity sensed along the Earth-vertical axis is less than the actual one. As a consequence, after the tilt, the component of the sensed velocity along the head-axis that was previously aligned with Earth-vertical is wrongly computed and the subject senses an overall rotation around an off-vertical axis that is not aligned with the axis of the actual rotation. It is important to remark that, although the tilt around the Earth-horizontal axis implies a re-orientation with respect to gravity, the axis of the ongoing rotation remains Earth-vertical in Coriolis/Cross-coupling. Therefore, no actual off-vertical axis rotation occurs and the orientation of the subject respect to gravity is fixed after the tilt, but the perceived rotation is equivalent to an off-vertical axis rotation not accompanied by the expected re-orientation with respect to gravity. In the train, a Coriolis/cross-coupling stimulus occurs due to the roll movements during both the acceleration and deceleration phases of yaw rotation (14). Cross-coupling occurring during high angular velocity rotations is one of the most powerful nauseogenic stimuli; whether the relatively low yaw velocities typical on curved train tracks (around 4°/s) are, however, adequate to cause motion sickness is not known.

It has been observed that if subjects’ heads were tilted during lateral acceleration, subjects had strong motion sickness, but if the head roll was initiated before the lateral acceleration, there was no motion sickness (28). By comparing different tilting systems in trains, it has been recently showed that an adequate synchronization of roll tilt with changes of yaw velocity on curves eliminates motion sickness during a 25-min travel on a curvy track (14). This could be expected if the sickness derives from the Coriolis/cross-coupling stimulus because the delay increases the drop in the sensed velocity caused by the high-pass filter behaviors of the semicircular canals. A larger drop of the sensed velocity before the tilt corresponds to a stronger miscalculation of the velocity component along the head-axis after the tilt (that was previously aligned with the Earth-vertical). This would imply a stronger Coriolis/cross-coupling, possibly causing more sickness. Nonetheless, other aspects of the stimulus in the study by Cohen et al. (14) changed concurrently with the change in tilt delay. This, together with the uncertainty on the strength of the Coriolis/cross-coupling stimulus at the low angular velocity involved prevented a definite conclusion on the cause of the sickness reduction. Moreover, the actual relevance of a delay in the tilt for such slow rotation is also unclear. The aim of this paper is to study the cross-coupling stimuli occurring in tilting train, isolating them from other motion stimuli to determine whether the phasing between the change of yaw velocity and the change of roll position is a decisive factor for tilting train motion sickness.

## Materials and Methods

### Study Cohort

Nineteen healthy subjects (nine females; mean age ± SD: 31 ± 9 years, range 22–66 years) participated in the study. Informed consent was obtained from all participants after full explanation of the experimental procedure. The protocol was approved by the Ethic committee of the Canton Zurich. In the recruiting phase, the subjects were requested to fill a questionnaire describing their previous experience on tilting train (Questionnaire Q0 in Supplementary Material). All included subjects had previous experience of traveling on tilting trains and declared to travel on a tilting train at least once per year. All subjects were naïve with respect to the apparatus used in the experiment.

### Experimental Setup

Subjects were seated upright on a turntable with three servo-controlled motor-driven axes (prototype built by Acutronic, Switzerland). The head was restrained with an individually molded thermoplastic mask (Sinmed BV, Reeuwijk, the Netherlands) to ensure that the movements matched those of the turntable. Subjects were positioned so that the intersection of the inter-aural and naso-occipital axes was at the intersection of the three axes of the turntable. A four-point safety harness secured the subjects. All experiments were performed in complete darkness.

### Experimental Protocol

#### Motion Stimuli

**Figure 1:**
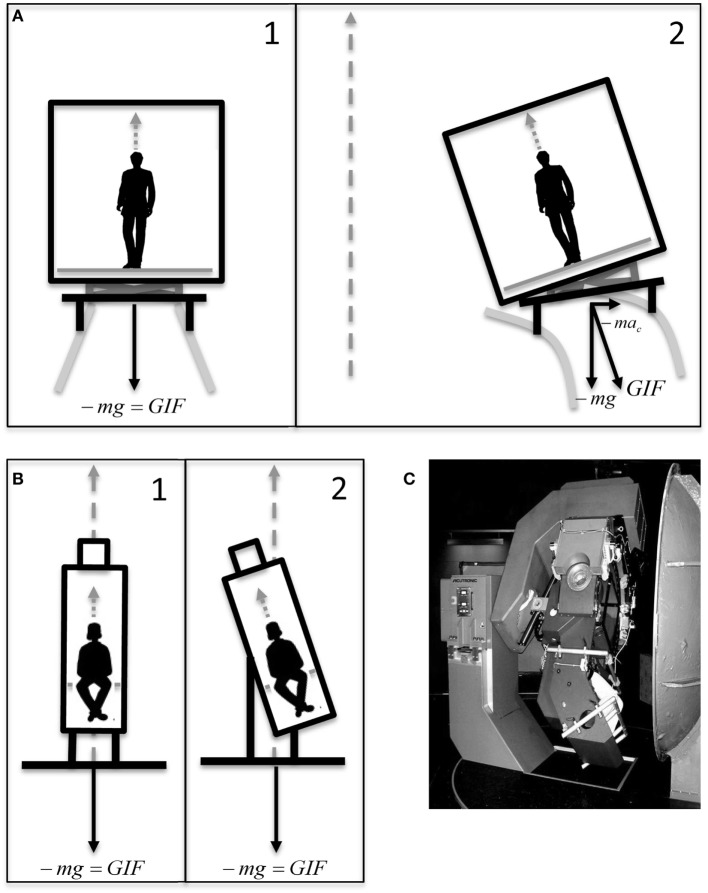
**(A)** Graphical representation of the motion of a car of a tilting train before (A1) and during (A2) a curve. The yaw axis of the curve (gray dashed arrow) does not coincide with the subject yaw axis before the tilt, since it passes through the center of the curve. This generates a centrifugal acceleration $\left(\vec{a_{c}}\right)$ tilting the gravito-inertial vector $\left(\vec{GIF}\right)$. The roll axis is below the car. **(B)** Graphical representation of the motion of the 3D turntable during our simulation of tilting trains before (B1) and during (B2) a simulated curve. The yaw axis of the turntable (gray dashed arrow) is aligned with the subject yaw axis before the tilt, and no tilt of the gravito-inertial vector $\left(\vec{GIF}\right)$ occurs. The roll axis is through the center of the head. Dotted gray arrows represent the yaw axis of the subject. **(C)** The 3D turntable.

For each subject, the experiment was split in two separate experimental sessions. Each experimental session consisted of two subsequent sequences of 12 simulated, ideal train curves each, reproduced using a 3D turntable (Figure 1C). The motion profile of the ideal curve was based on the data recorded from gyroscopes and accelerometers mounted on an actual train car during the experiment of the study by Cohen et al. (14). Since kinematics varied between the different curves recorded, parameters were adjusted to obtain an ideal curve based on the average values extrapolated from examples and graphs of Cohen et al. (14). Each simulated curve in our experiment consisted of a 30-s constant velocity yaw rotation with peak angular velocity of 4°/s and accelerations of 2°/s^2^. At the beginning of the curve, the subject was tilted 8° around the naso-occipital axis toward the inner part of the curve (i.e., toward right during rightward yaw rotation and left during leftward yaw rotation) and brought back upright at the end of the simulated curve (Figure 1B). The tilt profile had a peak velocity of 4°/s and acceleration of 10°/s^2^. This motion reproduced the misalignment between the body yaw axis and the rotation yaw axis occurring on tilting train. Different from the stimulus induced by a real train curve, the ideal curve did not reproduce the centrifugal acceleration (i.e., the gravito-inertial vector was not aligned with the body axis after the tilt, but remained aligned with the yaw axis of rotation). Moreover, the roll tilt axis of the simulated curve coincided with the naso-occipital axis of the subject, while in the tilting train is roughly 2 m below it (Figures 1A,B). After each curve, the subject was stationary for 20 s before a new curve was initiated. Each sequence contained six curves in clockwise direction and six in counterclockwise direction in randomized order. One entire session included, therefore, 24 simulated curves in 22–25 min, i.e., less than in the track described in Cohen et al. (14). All simulated rides were performed in total darkness.

**Figure 2:**
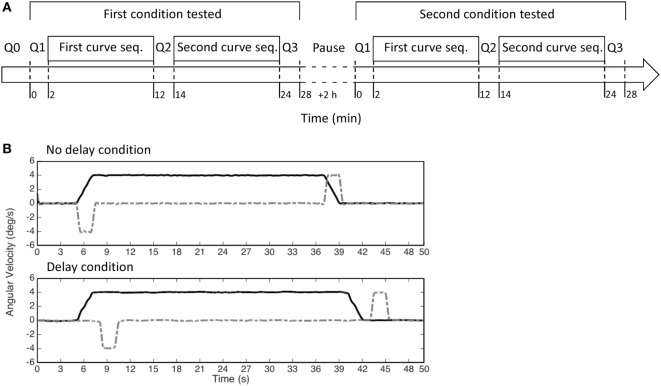
**(A)** Scheme of the experiment. Each experiment was divided in two sessions, which included two sequences of 12 curves each (first and second seq.). In each session, only one of the two conditions (i.e., no delay or delay) was tested, i.e., the two sequences of curves were identical. **(B)** The motion stimuli provided by our turntable in each curve (yaw velocity—black solid lines; roll velocity—gray dashed lines) in the no-delay condition (upper panel) and in the delay condition (lower panel).

After at least 2 h from the completion of the two sequences forming the first session, a second session was performed (Figure 2A). The two sessions were identical except for the timing of the tilt with respect to the onset of the curve. In one session, named “no-delay session,” the first tilt (at the beginning of the simulated curve) began at the same time of the yaw rotation and the second tilt, bringing the subject upright at the end of the curve, was initiated together with the deceleration of the yaw rotation. In the other session, named “delay session,” a delay of 3 s was added to the tilt with respect to the “no-delay” session (Figure 2B). The order of the two sessions was randomized across subjects and they were always performed within the same day.

#### Motion Sickness Evaluation

Before the experiment, subjects were instructed how to report motion sickness scores with a modified version of the simplified Pensacola motion sickness rating scale. This scale has been previously used in a number of studies and was proved effective for rapid reports of motion sickness score during the experiments (29–34). It requires the subject to rate the current feeling on a 0–20 scale, where 0 corresponds to “no reaction,” 5 to “starting to feel warm or have slight malaise,” 10 to “moderate gastro-intestinal distress and/or dizziness with or without sweating,” 15 to “strong feeling of nausea or dizziness, but the test could still be continued,” and 20 to “nausea/dizziness too strong to continue, vomiting.” In our modified version, we substituted the digital scale with analog scale (Questionnaire Q1–3 in Supplementary Material). The subjects were asked to rate their current feeling by marking a position along a 100-mm long vertical line, where the top of the line correspond to the 0 of the simplified Pensacola scale (i.e., no discomfort) and the bottom to the 20 (i.e., unable to continue the experiment). The position corresponding to 5, 10, and 15 of the simplified Pensacola scale were marked on the vertical line with short horizontal lines every 25 mm. The descriptions corresponding to the 0, 5, 10, 15, and 20 were provided in written form next to the line as a reference for the subject. The choice of the analog scale was motivated by the need to identify small variation of motion sickness scores and reduce the memory effects between subsequent reports, which we observed in pilot tests of the experimental protocol. The analog scale allows measuring variation of motion sickness score smaller than 1/20 (maximal sensitivity of the classical simplified Pensacola scale) without requiring the subject to use large numbers.

A questionnaire presenting the analog scale was provided to the subjects three times in each experimental session (Questionnaire Q1–3 in Supplementary Material): Q1—before the first 12-curves sequence; Q2—between the first and the second sequence; Q3—after the second sequence. A different questionnaire (Questionnaire Q0 in Supplementary Material) was provided to the subject in the recruiting phase, asking to report the frequency of their travel on tilting trains (from never to daily) and to rate the feeling induced by a typical ride on a tilting train using the same analog scale used in the experiment.

### Data Analysis

Questionnaires were analyzed by measuring the distance (millimeters) between the top of the line of the analog scale and the point marked by the subject. The number was rounded to the lower integer. This procedure transformed the 100-mm long line of the analog scale in a 0–100 simplified Pensacola scale. The values were imported into Matlab R2014b (Mathworks, USA) for further processing and statistical analysis. The differences D1 = Q2 − Q1 and D2 = Q3 − Q2 were calculated on a subject by subject basis to assess the variation of motion sickness due to the first and second sequence of curves separately. The resulting values were first grouped according to the order of session, obtaining D1_first_session_, D1_second_session_ and a D2_first_session_, D2_second_sesssion_, and then according to the stimulus condition (no-delay condition and delay condition) obtaining D1_no-delay_, D1_delay_ and D2_no-delay_, D2_delay_, respectively. Due to the crossover study design, all subjects were represented in all groups. Additionally, we calculated the values R1 = D1_delay_ − D1_no-delay_ and R2 = D2_delay_ − D2_no-delay_. These values express, for each subject, the differential increase of motion sickness score between the two conditions observed after the first (R1) and the second (R2) sequence, separately.

#### Statistical Analysis

Lilliefors test was used to assess the normality of the distribution of the difference vectors D1 and D2 for both data grouping strategies (no-delay/delay condition and first/second session). As the null hypothesis of normality was rejected, we presented the results using median and median absolute deviation [using the format: median (median absolute deviation)]. A Wilcoxon paired signed rank test was used to compare the values of D1 and D2 within and between the two conditions and the two sessions, separately. The Sperman correlation coefficient (ρ) was calculated between R1 and the results of the questionnaire Q0 and R2 and the results of the questionnaire Q0, computing the *p*-values for the estimated coefficients. To further verify whether the relative increase R1 of motion sickness due to the delay grows linearly with the sensitivity in tilting train Q0, a linear regression was calculated computing the *p*-values for the estimated coefficients and the *R*^2^ for the regression fits.

## Results

All subjects were able to complete the experiment in both conditions. Pooling data from both conditions, motion sickness scores recorded either after the first 12 curves of either condition (i.e., after the first sequence—Questionnaire B2) or at the end of each session (total of 24 curves, i.e., after the second sequence—Questionnaire B3) varied considerably ranging from 0/100 to 47/100. Similarly, the scores before starting the first sequence also showed considerable variations (0/100—39/100); in fact, some subjects were already feeling some unease before starting the experiment, but only one of them exceeded the score of 25/100, i.e., the first marked level on the analog scale (Questionnaire Q1–3 in Supplementary Material). The overall median (MAD = Median absolute deviation) motion sickness score recorded pooling all questionnaires of the three conditions were B1 = 0 [0], B2 = 4 [4] and B3 = 4 [4].

### Effect of the Order of the Sessions

Pooling the data of all subjects, we did not observe a significant difference of motion sickness scores (B Questionnaires) between the values reported before the first (B1_first_session_ = 0.5 [0.5]) and the before the second session (B1_second_session_ = 0 [0]) on the same day. By subtracting the motion sickness scores after each sequence from the one obtained before it, we computed the changes (D1, D2) in the score resulting from each of the two sequences of each session. Pooling the values based on the session order, no significant difference was found in the score change due to the first (D1_first_session_ = 1 [3]; D1_second_session_ = 0 [0.5]) or the second sequence (D2_first_session_ = 0 [2]; D2_second_session_ = 0 [1]) of the two sessions.

### Effect of the Simulation Condition

Different subjects reacted differently to the various sequence of curves, showing increases, decreases, or no variations of motion sickness scores in both conditions (Table 1). The median increase in motion sickness score during the first sequence in the delay condition was significantly higher (*p* = 0.04) than in the no-delay condition both in the pooled data of all subjects (D1_delay_ = 1 [4]; D1_no-delay_ = 0 [1]) and in the data of only the 12 subjects showing a variation in at least one of the two conditions (D1_delay_ = 8 [8]; D1_no-delay_ = 1.5 [4]). Moreover, D1_delay_ was found to have a median significantly different from 0 (*p* = 0.03), while it was not the case for D1_no-delay_.

**Table 1:** **Number of subjects showing either a decrease or an increase in motion sickness score after each sequence of 12 simulated curves**.

Sequence of curves	No delay		Delay		Pooled conditions	
Condition	First sequence	Second sequence	First sequence	Second sequence	First sequence	Second sequence
Increase in MS	6	9	10	8	12	14
Decrease in MS	3	3	2	2		
No change in MS	10	7	7	9	7	5

*Values are shown both divided by condition and pooled*.

The second sequence did not led to increases with a median different from 0 (D2_delay_ = 0 [4]; D2_no-delay_ = 0 [2.5]) in any conditions, and no significant difference was found between the increases due to the second sequence when comparing the two conditions. These results were confirmed even when considering only the 14 subjects changing their motion sickness score during to the second sequence in at least one condition (D2_delay_ = 4 [4]; D2_no-delay_ = 1 [2.5]).

### Correlation with Motion Sickness Experienced in Tilting Train

**Figure 3:**
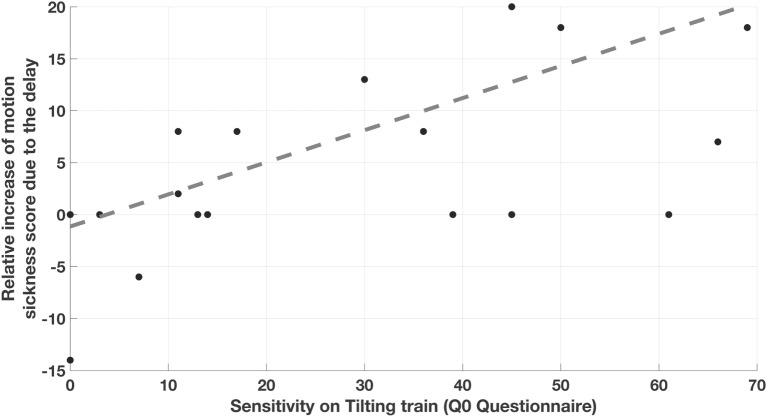
**Correlation between the relative increase of motion sickness score caused by the delay and the motion sickness sensitivity experienced on previous tilting train rides**. Each point correspond to one subject and report on the *y*-axis the difference between the increases in motion sickness score caused by the first sequence of curves with 3 s of delay (D1_delay_) and the one caused by the sequence with 0 s delay (D1_no-delay_).

A significant (*p* = 0.003, ρ = 0.65) correlation was found between the variation D1_delay_ due to the first sequence of curves in the delay condition and the motion sickness score reported to describe the previous experience on tilting trains (Q0). This was, however, not the case for the variation D1_no-delay_ resulting from the first sequence of curves in the no-delay condition. The relative increase R1 of motion sickness score as a result of the delay (Figure 3) also significantly correlate with Q0 (*p* = 0.01, ρ = 0.58). As R1 was obtained by subtracting the increases in the no-delay condition from those in the delay condition within each subject, this results further evidence that the delay in the tilt is the factor leading to a correlation of the recorded score with Q0. A linear regression of D1_delay_ and R1 with respect to the score experienced on tilting trains (Q0) confirmed the above correlations (*p* = 0.004, *R*^2^ = 0.40 and *p* = 0.008, *R*^2^ = 0.35 for D1_delay_ and R1, respectively), supporting the hypothesis of a linear relation between the variables. No correlation was observed between the further increases caused by the second sequence of curves neither considering the two conditions alone, or the relative increase R2.

## Discussion

Our results demonstrate that the Coriolis/cross-coupling stimuli can induce motion sickness even when the velocity of the yaw rotation is relatively low, as it occurs during train travels (14). This confirms the hypothesis of the previous study performed during an actual train ride (14), which suggested that Coriolis/cross-coupling caused by the delay in the tilt of the train cars was one of the key causes of motion sickness in tilting trains. In that experiment, however, jitters of the cars, as well as lateral accelerations due to centripetal forces, could have played a role in determining the subjects’ responses. In our experiment, no additional linear translation due to the centrifugal force were present (the turntable yaw axis was aligned with the subject midline), and the conflict between motion stimuli only depended on the misalignment between the perceived direction of gravity and the perceived rotation axis, i.e., we tested the effect of a pure Coriolis/cross-coupling stimuli induced by the angular rotation motion profile typical of tilting train during a very short ride (24 curves).

The Coriolis/cross-coupling stimulus was only nauseogenic when the tilt of the chair was delayed with respect to the beginning of the constant velocity rotation (i.e., the simulated curve). The 3-s used in this simulation exaggerated the delay observed in the tilting train (14). This difference, however, allowed to clearly discriminate between the two conditions tested and to evidence that the delay is the relevant variable causing Coriolis/cross-coupling dependent motion sickness in tilting train. The no-delay condition, indeed, did not induce any change in the median motion sickness in our tested subject.

Motion sickness was significantly stronger in the most sensitive subjects. Instead of assessing general sensitivity to motion sickness (35), we decided to collect information specifically oriented on the recent experience on tilting train, with the aim of understanding the contribution of tilt delay to the specific motion stimulus. The self-assessed sensitivity to tilting trains did correlate significantly with the motion sickness observed in the delay condition but not in the no-delay condition. The lack of correlation found in the no-delay condition implies that even those of our subjects showing the highest train-specific sensitivity did not suffer from significant increases of symptoms when no delay was present. Although with a lower correlation coefficient than the non-parametric correlation, the significant correlation found with the linear regression (Figure 3) between the relative increase of motion sickness due to the delay and the self-assessed sensitivity supports the hypothesis that our simulation reproduces a portion of the sickness experienced in the train, i.e., tilt delay is a specific trait of the motion stimulus causing disturbances to the subjects complaining for motion sickness on tilting trains.

The present experiment, by isolating the Coriolis/cross-coupling stimuli occurring in the train from all others stimuli present in the study of Cohen et al. (14), clearly demonstrates that, even at angular velocity as low as 4°/s, a small tilt (i.e., the cause of the Coriolis/cross-coupling stimulus) is nauseogenic if it is delayed with respect to the rotation onset. It can be argued, however, that our study does not prove that the cross-coupling stimulation with delay is the major factor causing sickness during actual rides. Only a concurrent series of test, isolating each stimulus present in the actual rides, could indeed provide the exact weight of each component. With respect to this, it is interesting to consider that, when the delay was present, motion sickness rose rapidly in our subjects, reaching a stable level within the first 12 curves (10 min) and did not increase further. The nauseogenic effect that we observed is, therefore, unlikely to be preceded by that of other motion stimuli caused by the train. The high motion sickness scores reported in such short time by the subjects with the highest train-specific susceptibility suggest that the delay in the tilt of the car provide a relevant contribution to the overall motion sickness experienced by passengers. For a thorough comparison, however, two aspects of our simulation need to be considered. First, the simulated curves of the delay condition exaggerated the tilt delay to magnify the difference between the conditions and were likely more provoking than the curve of the train. Second, our simulation included less curves than the train ride tested by Cohen and colleagues (14), but the overall duration was similar, i.e., the frequency of nauseogenic stimuli was lower than on the train, and therefore possibly less nauseogenic. Combining these two differences between our simulation and an actual train ride (14), it is difficult to draw a conclusion on the relative role of the tilt based on the intensity of the nauseogenica, and we cannot exclude that the other nauseogenic stimuli still contribute to the motion sickness and, if tested in isolation, could generate high level of motion sickness in a similarly short time.

Although only additional experiments testing the linear stimulations in isolation could provide a definitive answer on the relative role of each component, we can conclude that even the low angular velocities of the tilting train generate cross-coupling stimuli capable of causing high motion sickness. Cross-coupling is, therefore, a relevant contributor to motion sickness in tilting train. More interesting, we further evidence that the effect of cross-coupling is almost nulled by reducing the delay, as suggested by Cohen et al. (14).

## Ethics Statement

This study was carried out in accordance with the recommendations of Ethic Committee of the Canton Zurich with written informed consent from all subjects. All subjects gave written informed consent in accordance with the Declaration of Helsinki.

## Author Contributions

GB and DS conceived the study, designed the experiment, critically contributed to interpretation of the results and draw the conclusions, and wrote the manuscript. GB analyzed the data and performed the statistic. MD, KF, CL, and AK refined the experimental design, performed the experiment, acquired and analyzed the data, and interpreted the results.

## Conflict of Interest Statement

The authors declare that the research was conducted in the absence of any commercial or financial relationships that could be construed as a potential conflict of interest. The reviewer, SY, and handling editor declared their shared affiliation, and the handling editor states that the process nevertheless met the standards of a fair and objective review.
