# Supplementary material for: Determinants of Motion Sickness in Tilting Trains: Coriolis/Cross-Coupling Stimuli and Tilt Delay
Source: Front Neurol. 2017 May 15;8:195. doi: 10.3389/fneur.2017.00195 (PMC5430385; doi:10.3389/fneur.2017.00195)
Supplement: Supplementary file 1 [file Data_Sheet_1.DOCX]

| **Participant’s number** | | | | | \|  \|  \|  \| \| --- \| --- \| --- \| \|  \|  \|  \| | | |
| --- | --- | --- | --- | --- | --- | --- | --- | --- | --- | --- | --- | --- | --- |
| **Did you take anything for motion sickness today?** (e.g. pills, drops, globules, etc.) | | | | | | | |
| 🞎 no | | | | | |  | |
| 🞎 yes, what: at what time?: | | | | | | \|  \|  \| : \|  \|  \| \| --- \| --- \| --- \| --- \| --- \| \|  \|  \|  \|  \| | |
| **How often do you travel with a tilting train on average?** | | | | | | | |
| never | very rarely | 1x yearly | 1x monthly | 1x weekly | | | 1x daily |
| 🞎 | 🞎 | 🞎 | 🞎 | 🞎 | | | 🞎 |

**If more than “very rarely”: How do you judge your physical health during a ride on a tilting train?**

normal health, no discomfort

slight malaise (light discomfort, starting to feel warm)

moderate malaise (discomfort in the gastro-intestinal tract and/or dizziness with/without sweating)

strong malaise (dizziness or nausea but could continue traveling)

dizziness is too strong to continue traveling, vomiting

**That’s it. Thank you very much!**

| **Participant’s number** | \|  \|  \|  \| \| --- \| --- \| --- \| \|  \|  \|  \| |
| --- | --- | --- | --- | --- | --- | --- | --- |

**How do you judge your physical health now? (Please avoid considering discomfort caused by the mask, belt and chair)**

normal health, no discomfort

slight malaise (light discomfort, starting to feel warm)

moderate malaise (discomfort in the gastro-intestinal tract and/or dizziness with/without sweating)

strong malaise (dizziness or nausea but could continue with the test)

dizziness is too strong to continue the test, vomiting

**That’s it. Thank you very much!**
